# Enhanced crystalline cellulose degradation by a novel metagenome-derived cellulase enzyme

**DOI:** 10.1038/s41598-024-59256-4

**Published:** 2024-04-12

**Authors:** Faezeh Kholousi Adab, Mohammad Mehdi Yaghoobi, Javad Gharechahi

**Affiliations:** 1 Department of Biotechnology, Institute of Science and High Technology and Environmental Sciences, Graduate University of Advanced Technology, Kerman, Iran; 2 Human Genetic Research Center, Baqiyatallah University of Medical Sciences, Tehran, Iran

**Keywords:** Metagenome, Cellulase, Endoglucanase, Exoglucanase, Bioprospecting, Avicelase, Acidophilic, Applied microbiology, Environmental biotechnology

## Abstract

Metagenomics has revolutionized access to genomic information of microorganisms inhabiting the gut of herbivorous animals, circumventing the need for their isolation and cultivation. Exploring these microorganisms for novel hydrolytic enzymes becomes unattainable without utilizing metagenome sequencing. In this study, we harnessed a suite of bioinformatic analyses to discover a novel cellulase-degrading enzyme from the camel rumen metagenome. Among the protein-coding sequences containing cellulase-encoding domains, we identified and subsequently cloned and purified a promising candidate cellulase enzyme, Celcm05-2, to a state of homogeneity. The enzyme belonged to GH5 subfamily 4 and exhibited robust enzymatic activity under acidic pH conditions. It maintained hydrolytic activity under various environmental conditions, including the presence of metal ions, non-ionic surfactant Triton X-100, organic solvents, and varying temperatures. With an optimal temperature of 40 °C, Celcm05-2 showcased remarkable efficiency when deployed on crystalline cellulose (> 3.6 IU/mL), specifically Avicel, thereby positioning it as an attractive candidate for a myriad of biotechnological applications spanning biofuel production, paper and pulp processing, and textile manufacturing. Efficient biodegradation of waste paper pulp residues and the evidence of biopolishing suggested that Celcm05-2 can be used in the bioprocessing of cellulosic craft fabrics in the textile industry. Our findings suggest that the camel rumen microbiome can be mined for novel cellulase enzymes that can find potential applications across diverse biotechnological processes.

## Introduction

Most herbivorous animals, particularly ruminants, depend on a complex microbial community in their gastrointestinal tract to digest and utilize plant-based fiber as the sole nutrient source. This relationship is obligatory and symbiotic because host animals lack the hydrolytic enzymes necessary to break down the complex lignocellulosic structure of plant cell walls. Ruminant animals have developed a specialized stomach organ called the rumen, which serves as a large fermentative chamber. It provides an optimal environment characterized by stable pH, temperature, and anaerobic conditions, facilitating the colonization of a diverse community of microorganisms from archaea, bacteria, and eukaryotes . The rumen enables ruminants to store plant material and provides the necessary conditions and time for microbial-based digestion and fermentation of plant fiber. The degradation process is aided by the formation of a dense biofilm on the surface of feed particles by lignocellulose-degrading bacteria^1^. While attached to feed particles, rumen bacteria produce a complex cocktail of substrate- or linkage-specific carbohydrate-active enzymes to efficiently break down resilient cellulose, hemicellulose, and pectin polysaccharides present in the plant cell wall^2^.

Traditionally, the mining of polysaccharide-degrading enzymes from rumen microorganisms was constrained to those microorganisms capable of thriving under in vitro cultivation conditions. However, this approach had limitations as it overlooked a substantial portion of rumen microorganisms that could potentially possess crucial enzymatic mechanisms for effective fiber digestion, as many of these microorganisms were not amenable to isolation and cultivation in pure culture^3^. The advent of culture-independent approaches has provided us with a more comprehensive understanding of the polysaccharide-degrading potential harbored by rumen microorganisms^2,4^. These innovative methods facilitate two distinct avenues: the direct cloning of DNA extracted from rumen samples into surrogate hosts like *E. coli* for subsequent activity-based screening for potential polysaccharide-degrading enzymes (functional metagenomics)^4–7^, or sequencing the total metagenome DNA to identify protein-coding genes (shotgun metagenomics). The latter enables in silico prediction and annotation of lignocellulose-degrading enzymes in metagenome sequencing data^3,8^. In recent years, numerous polysaccharide-degrading enzymes have been characterized through the metagenome sequencing^9–12^.

The recent metagenome sequencing of rumen microbiota has yielded an abundance of sequences encoding polysaccharide-degrading enzymes, presenting a vast array of potential candidates with catalytic properties suitable for industrial applications^2,13–15^. Nevertheless, identifying specific enzymes with novel functional properties remains challenging amidst the redundancy of enzymes predicted through metagenome sequencing. To address this challenge, in silico analyses, including sequence homology search, phylogenetic analysis, three-dimensional structure predictions, and combinatorial approaches utilizing machine-learning methods, have proven helpful^3,16^. These approaches aid in the shortlisting and prioritizing of sequences, enabling targeted experimental characterization of the enzymes of interest. Notably, a recent development includes the application of a machine learning-based method to differentiate between xylanase encoding sequences based on their thermostability properties^17^. In an effort to streamline the process of discovering carbohydrate-active enzymes (CAZymes), an automated search tool called SACCHARIS was developed^18^. SACCHARIS allows for targeted searches of complex genomic or metagenomic datasets utilizing sequence and structural features, enabling the identification of enzymes with novel carbohydrate-degrading or binding activity.

The CAZy database categorizes cellulase enzymes into distinct families based on their sequence, folding, and specific activity^19^. Cellulases play a significant role in the degradation of plant-based fiber within the rumen. These enzymes are predominantly produced by small lineages of rumen microbiota specifically adapted to efficiently break down this resilient polysaccharide^1^. The breakdown of cellulose represents a rate-limiting step in the process of plant cell wall degradation, as this insoluble polysaccharide forms hydrogen-bonded crystalline microfibrils that are inaccessible to enzymatic hydrolysis^20^. The complete hydrolysis of cellulose necessitates the synergistic action of three different types of enzymes: endoglucanases (EC 3.2.1.4), exoglucanases (or cellobiohydrolases, EC 3.2.1.91), and β-glucosidases (EC 3.2.1.21). Endoglucanases act on internal β-(1–4) glycosidic linkages, while exoglucanases hydrolyze cellulose chains from both the reducing and non-reducing ends, releasing cello-oligosaccharides (2–4 units) that are subsequently processed by β-glucosidases into glucose monomers^21^.

Cellulases find extensive application in diverse industrial processes that demand enzymatic hydrolysis of cellulose, such as biofuel production, paper and pulp manufacturing, animal feed production, detergent formulation, and textile processing^22,23^. There is significant interest in bioprospecting cellulases with enhanced catalytic properties, such as higher activity and the capability to retain their functionality under extreme thermal and pH conditions^24^. Additionally, there is a growing demand for cellulases that exhibit sustained activity in the presence of high salt concentrations, detergents, organic solvents, and even ionic liquids^25,26^. These desirable traits would greatly expand the utility and versatility of cellulases in various industrial applications and biotechnological processes.

In this study, we utilized sequence homology search and phylogenetic analysis to identify a glycoside hydrolase belonging to family 5 (GH5) within the previously described metagenome of the camel rumen^27^. The enzyme was cloned and purified to homogeneity for characterization under different substrate and reaction conditions.

## Materials and methods

### Sequence analysis

Animal rumen sampling, metagenome sequencing, assembly, and annotation have been described elsewhere^27^. Protein coding genes were predicted using Prodigal (v.2.6.3) in the metagenome mode^28^. CAZymes were annotated using profile HMMs from the CAZy database (v.11) using run_dbcan (v.3.0.7), considering a *p*-value cutoff of 1e-15 and coverage of 0.35^29^. A total of 203 full-length cellulase-encoding genes belonging to families GH5, GH9, GH45, and GH48, were selected based on the annotations obtained from the run_dbcan search. To further analyze these candidate sequences, a search was conducted against the NCBI non-redundant (nr) protein database using diamond (v.2.0.13)^30^, with an e-value cutoff of 1e-5 and a maximum of 30 target sequences (–max-target-seqs 30). Taxonomies were assigned to the sequences using the lowest common ancestor algorithm in MEGAN, which provides insights into the conservation level of the sequences^31^. Sequences containing a signal peptide (secretion signal) were identified using Signalp (v.4.1) operating in both Gram-positive and Gram-negative modes. The candidate sequence was finally chosen from a subset of enzyme sequences that possessed a secretion signal and originated from lignocellulose-degrading bacteria found in the rumen. The most distantly related sequences to the candidate sequence were identified using PSI-BLAST (7 rounds), and those with a minimum sequence identity of 45% were selected for dereplication (ensuring only one representative sequence per species). The dereplicated sequences were used for multiple sequence alignment and phylogenetic tree construction. The phylogenetic tree was constructed in MEGA software v.11.0.13^32^ using the Maximum Likelihood method with the LG with Freqs. (+ F) model and the analysis was performed with 999 bootstrap replications.

### PCR amplification and cloning

The gene encoding the candidate enzyme (CelCm05-2) was amplified from a DNA sample previously utilized for metagenome sequencing^33^, using the primers 5′-ATGC**CCATGG**GAGTATCTGGTAGTGCGCATC-3’ (NcoI site is underlined) and 5'-ATGC**AAGCTT**AAGCATTGCTTTCATTATATCAGGA-3’ (HindIII site is underlined). The forward primer was specifically designed to amplify from an internal site, excluding the secretion signal peptide. A touchdown PCR protocol was employed to enhance amplification specificity from metagenomic DNA. The protocol commenced with an initial 10 cycles, where the annealing temperature was gradually decreased by 0.5 ℃ per cycle from 65 ℃, followed by 25 cycles at a constant temperature of 60 ℃. The amplified DNA fragment (1250 bp) was gel purified and digested with NcoI and HindIII restriction enzymes (Thermofisher, Massachusetts, USA) and subsequently ligated into a NcoI-HindIII linearized pET28a vector using T4 DNA ligase (Thermofisher). The construct was confirmed by Sanger sequencing.

### Expression and purification of the recombinant enzyme

The vector carrying the cellulase gene was transformed into a chemically competent *E. coli* BL21 (DE3) expression host. The transformed cells were cultivated in lysogeny broth (LB) media supplemented with 50 µg/mL kanamycin at 37 ℃. Once the culture reached an optical density of 0.5 at 600 nm (OD_600_), 0.2 mM of isopropyl-β-thiogalactopyranoside (IPTG) was introduced, and the culture was subsequently incubated overnight for 18 h at 18 ℃ in a shaking incubator at 200 rpm. Cells were harvested by centrifugation at 4000 × g for 10 min, resuspended in a lysis buffer (50 mM NaH_2_PO_4_, 300 mM NaCl, and 10 mM imidazole, pH 8.0) supplemented with 1 mM PMSF and 0.2 mg/mL lysozyme and incubated for 30 min on ice. The cells were disrupted by sonication on ice using 60% amplitude and 10-s pulses (10 times with 30-s intervals). The cell lysate was centrifuged at 10,000 × g and 4 ℃ for 30 min. The cleared lysate was collected and applied to a NI-NDA agarose resin column (Pars Tous, Iran), followed by a 60-min incubation on ice. Subsequently, the buffer was drained, and the column was washed at least two times with wash buffer (50 mM NaH_2_PO_4_, 300 mM NaCl, and 20 mM imidazole, pH 8.0). The His-tagged protein was then eluted using elution buffer (50 mM NaH_2_PO_4_, 300 mM NaCl, and 250 mM imidazole, pH 8.0), and the resulting protein fraction was concentrated to eliminate excess imidazole using an Amicon® Ultra-4 centrifugal filter with a 30 kDa cutoff (Millipore, Germany). The protein was recovered in a storage buffer containing 50 mM Tris–HCl pH = 8, 150 mM NaCl, 1 mM DTT, and 50% glycerol. The protein’s purity was evaluated through sodium dodecyl sulfate–polyacrylamide gel electrophoresis (SDS-PAGE). The protein concentration was determined using the Bradford assay, with bovine serum albumin used as the standard, in a microplate reader (Bio-Rad, Carlsbad, USA).

### Enzyme activity assay

The activity of the recombinant enzyme was initially assessed using an agar plate (1% in phosphate buffer, pH = 7) supplemented with 0.1% carboxymethylcellulose (CMC, Molekula, England) as the substrate. To create holes in the agar plate, small iron balls measuring 4 mm in diameter were placed on the surface of the solidifying agar plate. Subsequently, the iron balls were removed using a magnet, and the enzyme solution (20 µL diluted solution) was carefully loaded into the resulting holes. The plate was incubated at 37 ℃ for 20 h. Subsequently, the plate surface was covered with 0.1% (w/v) Congo red solution (Biobasic, Ontario, Canada) for 15 min, followed by washing with a 1 M NaCl solution for 15 min. The diameter of the cleared zone surrounding the perforation reflects the enzyme’s hydrolytic activity.

### Determining optimal pH and temperature

The enzyme’s optimal pH was determined by measuring its activity across a pH range of 2–10, employing different buffering systems. pH 2 was assayed using a glycine–HCl buffer, pHs 3–6 were assessed using a 50 mM citrate buffer, pHs 7–8 were evaluated with a 50 mM phosphate buffer, and pHs 9–10 were examined using a 50 mM glycine–NaOH buffer. Enzyme activity was measured at different pH values using CMC as substrate. All assays were conducted in triplicate.

To determine the optimal temperature for Celcm05-2, activity measurements were taken over a temperature range of 10 to 80 ℃. Since the enzyme exhibited high activity at pH 3, the optimal temperature was evaluated at this pH. The reaction was performed in triplicate using CMC as the substrate, following the same procedure described for the CMCase assay.

### Substrate specificity of Celcm05-2

#### CMCase activity

CMCase activity was assayed using 1% (w/v) CMC solution as the substrate in 50 mM citrate buffer at pH 3, following the previously described method^34^. The reaction mixture consisted of 55 µL of the CMC substrate in citrate buffer and 5 µL of the enzyme solution. The reaction was conducted at 40 ℃ for 30 min in a water bath. The amount of reducing sugar released through CMC hydrolysis was measured at a wavelength of 540 nm by the 3,5-Dinitrosalicylic acid (DNS) method^35^, with a glucose standard of 2 mg/mL. One Unit of the enzyme was defined as µmols of reducing sugar released per minute per milligram (µmol min^−1^ mg^−1^) or per milliliter (µmol min^−1^ mL^−1^) of the enzyme.

#### Filter paper assay (FPase)

The FPase assay followed a similar procedure to the CMCase assay, substituting the substrate for the Whatman No. 1 filter paper. Filter papers were prepared by cutting them into 6 mm diameter disks using an office paper puncher. Each filter paper disk was immersed in 55 μL of 50 mM citrate buffer at pH 3, along with 5 μL of the enzyme solution, as previously described^36^. The reaction mixture was then incubated at 40 ℃ in a water bath for 1 h. The quantity of reducing sugars released was determined using the DNS method.

#### Avicel

The enzyme’s activity on crystalline cellulose (Avicel) as a substrate (1% w/v) was also evaluated. The reaction was conducted using three independent replications, following the same procedure described for the CMCase assay, except the reaction time was extended to 1 h.

#### Xylan

The xylanase activity of Celcm05-2 was measured in the presence of 1% xylan (Molekula). The reaction was conducted in triplicate and the amount of xylose released was measured using a xylose standard curve.

#### Effect of salt, organic solvents, metal ions, and inhibitors on enzyme activity

The activity of Celcm05-2 was assessed to evaluate its tolerance to high salt concentration by performing the assay in the presence of 3 M NaCl. The enzyme activity was measured after 20 h of salt exposure. Furthermore, the effect of various metal ions, including Mg^2+^, K^+^, Mn^2+^, Ni^2+^, and Ca^2+^, at concentrations of 2 and 10 mM, was examined under the same conditions as the CMCase assay. The impact of various organic solvents, including isopropanol, methanol, ethanol, chloroform, and glycerol, at a concentration of 30% (v/v), on enzyme activity was also determined. Additionally, the influence of enzyme inhibitors such as PMSF and EDTA, as well as detergents like SDS and Triton X-100, and reducing agents, including dithiothreitol (DTT), was also assessed. All assays were performed in triplicate at a temperature of 40 ℃ in a water bath.

#### Saccharification of the waste carton by Celcm5-2 enzyme

The waste paper cartons were cut into small pieces and soaked in distilled water for 2 h. After soaking, the carton pieces were ground in a grinder, and the resulting pulps were dried in an oven at 50 ℃. Subsequently, the dried pulps were soaked in 50 mM citrate buffer (pH 3) and treated with the enzyme (5 µL) for 192 h at 40 ℃ in a water bath. As a control, a parallel reaction without the enzyme was also maintained. The amount of released sugar was measured using the DNS method. The structural changes in the paper pulps were evaluated using scanning electron microscopy (SEM, VEGA3, TESCAN) and Fourier transform infrared spectroscopy (FTIR, BRUKER).

#### Determination of kinetic parameters

To determine the enzyme kinetic parameters, the activity of Celcm05-2 was measured in the presence of a range of CMC concentrations from 1 mg/mL to 10 mg/mL in a 50 mM citrate buffer (pH 3) and at a temperature of 40 ℃ in a water bath. The Michaelis constant (K_m_), turnover number (K_cat_), and maximum reaction rate (V_max_) were calculated using the nonlinear regression method in R statistical software.

#### Predicting enzyme three-dimensional (3D) structure and in silico structural analysis

The 3D structure of the protein was predicted using Alphafold2^37^. The most similar 3D structure to the enzyme in the PDB database was also identified using the I-TASSER server^38^. The AlphFold2 predicted structure was aligned and superimposed onto a similar crystallographic PDB structure using ChimeraX software v.1.6.1^39^. The protein’s physicochemical properties were predicted using the ProtoParam web server (https://web.expasy.org/protparam/). Residues contributing to the enzyme’s active site were predicted using the COFACTOR web server^40^.

### Statistical analysis

All statistical analyses were performed in R statistical software. Enzyme activity under various substrate or reaction conditions was analyzed using one-way analysis of variance (ANOVA), and means were compared using the Tukey post hoc test. A *p*-value < 0.05 was considered statistically significant.

## Results

### In silico analysis revealed that Celcm05-2 is a novel family 5 glycoside hydrolase

Celcm05-2 was chosen from a compilation of GHs discovered in the metagenome of fiber-attached microbiota in the camel rumen^27^. The candidate sequences underwent analysis using different search tools to assess their taxonomic origins and catalytic capabilities in polysaccharide degradation. Phylogenetic analysis confirmed that Celcm05-2 is derived from a *Butyrivibrio* species, a recognized lignocellulose-degrading phylotype in the rumen. Celcm05-2 was predicted to possess a lipoprotein secretion signal peptide (Sec/SPII) within the initial 16 residues. Proteins harboring this signal are typically transported through the Sec translocon and subsequently cleaved by signal peptidase II.

**Figure 1:**
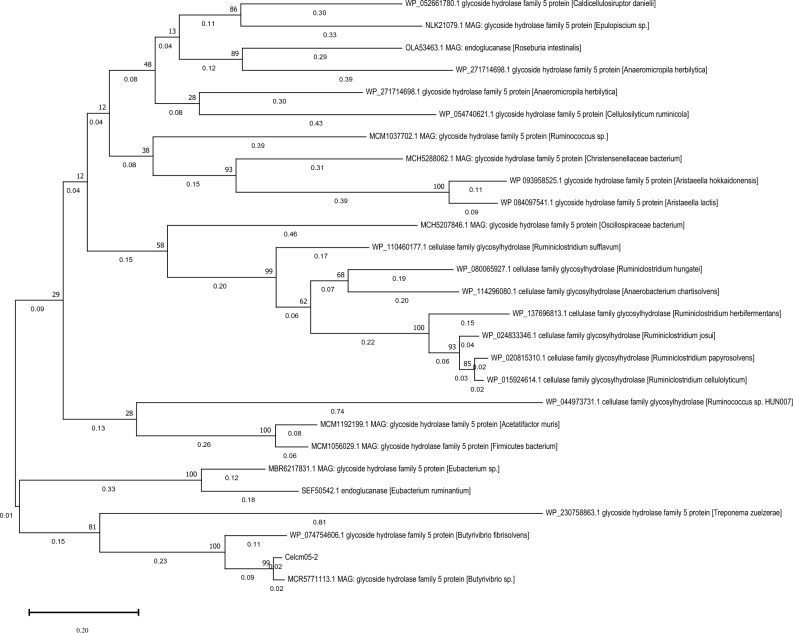
Phylogenetic analysis of Celcm05-2. The phylogenetic tree shows the relationship of Celcm05-2 with the most similar sequences deposited in the NCBI nr database. The sequences were identified using 7 rounds of position-specific iterative BLAST (PSI-BLAST). The phylogenetic tree was constructed using the maximum likelihood method considering 999 bootstrap replications.

According to a BLASTP search, Celcm05-2 exhibited the highest sequence identity (96%) with a GH5 derived from *Butyrivibrio sp*. previously identified in a metagenome-assembled genome reconstructed from the camel rumen metagenome^15^. The second most similar sequence (79%) corresponded to a GH5 glucanase (accession number: WP_074754606.1) from the well-known bacterium *B. fibrosolvens*, for which an AlphFold-predicted 3D structure (A0A1H9NA26_A) is available. The phylogenetic analysis consistently grouped Celcm05-2 with these sequences and distinguished it from representative sequences of other taxa, indicating its limited sequence similarity to known sequences in the database (Fig. 1). Searching for conserved domains in Celcm05-2 revealed the presence of a 291 amino acid Glyco_hydro superfamily domain (Pssm-ID: 451508) spanning residues 117–408 with an e-value 1.81e-64. The Hidden Markov Model (HMM)-based analysis classified Celcm05-2 as a member of subfamily 4 within the glycoside hydrolase family 5 (GH5_4). Members of this subfamily are typically known for their xyloglucan-specific endo-β-1,4-glucanase, (EC 3.2.1.4 and EC 3.2.1.151), licheninase (EC 3.2.1.73), and xylanase (EC 3.2.1.8) activities^41^.

### Physicochemical properties and 3D structure of Celcm05-2

The open reading frame of the Celcm05-2 gene was predicted to encode a 438 amino acid length protein with a theoretical pI of 4.33 and a molecular weight of 49 kDa. Celcm05-2 was predicted to be a stable protein with an instability index of 33.2. It also exhibits an aliphatic index of 81.3 and a Grand average of hydropathicity (GRAVY) of − 0.422, indicating its potential thermostability and hydrophilicity.

**Figure 2:**
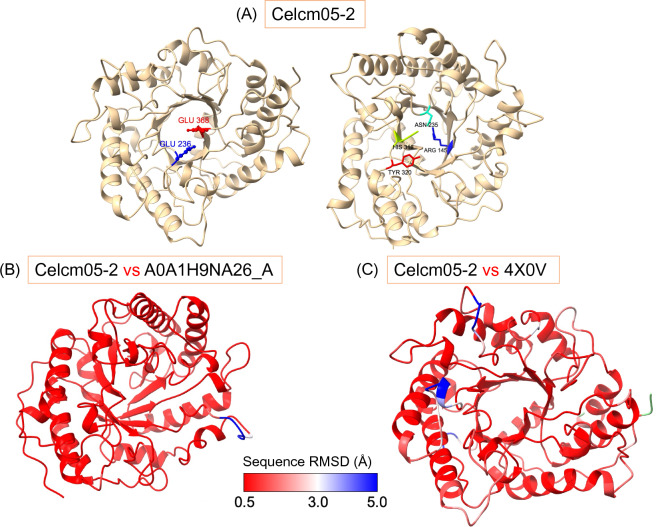
The three-dimensional structure of Celcm05-2. (**A**) The 3D structure of the recombinant protein was predicted using Alphafold. The first 70 unstructured residues are not shown. The residues constituting the active site cleft have been marked with numbers showing their position in the original protein sequence. The two Glu residues (Glu236 and Glu368) constitute the catalytic proton donor and the nucleophile acceptor. A comparison of the 3D structure of Celcm05-2 with the predicted 3D structure of most similar glycoside hydrolase sequences deposited in the NCBI database (**B**) and the crystallographic structure of GH5 lichenase obtained from Caldicellulosiruptor sp. F32 (**C**). Only the structure of Celcm05-2 is shown. Residues are colored based on root-mean-square deviation (RMSD) values in two superimposed structures in Angestrum (Å). Missing residues in alignment are colored in light green.

The 3D structure of Celcm05-2 was predicted using AlphaFold, revealing a distinct (β/α)_8_ barrel-shaped structure characteristic of the GH5 CAZyme family (Fig. 2A). Further threading analysis identified the 4X0V structure as the most similar 3D structure to Celcm05-2 in the PDB database. The 4X0V structure corresponds to a GH5 lichenase from *Caldicellulosiruptor* sp. F32 determined through X-ray diffraction at a resolution of 2.80 Å^42^. Comparing the predicted structure of Celcm05-2 with the AlphaFold predicted structure of WP_074754606.1 (displaying 79% sequence identity with Celcm05-2), which encodes for a GH5 glucanase, and the 4X0V structure revealed an overall similar folding pattern indicating their shared catalytic properties (Fig. 2B,C). All three structures exhibit a common feature—an internal barrel formed by 8 β-strands, which is enveloped by 8 α-helices, effectively encasing the active site cleft.

**Figure 3:**
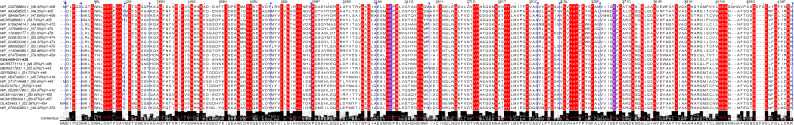
Multiple sequence alignment of Celcm05-2 with distantly related sequences in the NCBI database. Conserved residues are highlighted in red, while the two catalytic glutamic acid residues (Glu236 and Glu368) forming catalytic dyads are shaded in purple. The residues have been numbered following the Celcm05-2 sequence. Vertical blue lines show the positions of hidden residue columns.

A multiple sequence alignment of Celcm05-2 with distantly related sequences, identified through PSI-BLAST, revealed the presence of several conserved residues that likely play a crucial role in catalysis (Fig. 3). Notably, two glutamic acid residues (E236 and E368) within the active site cleft appear to act as the catalytic proton donor and nucleophile, typical of this family of CAZymes^42^. Furthermore, other residues in this region, including R145, N235, H318, and Y320, are highly conserved across diverse sequences (Fig. 3), highlighting their importance in substrate binding and the enzyme’s catalytic activity.

### Cloning, expression, and purification of the recombinant Celcm05-2

Celcm05-2 was cloned in the pET28a expression vector and expressed heterologously in the *E. coli* BL21 (DE3) expression host. A His-tag was introduced at the C-terminal end of the recombinant protein to facilitate easy purification, allowing for one-step purification through the NI–NTA affinity chromatography procedure. Subsequent SDS-PAGE analysis of the purified protein confirmed the presence of a single band, aligning with the expected molecular weight (~ 50 kDa) of Celcm05-2 (Fig. S1A).

### Enzymatic properties of Celcm05-2

**Figure 4:**
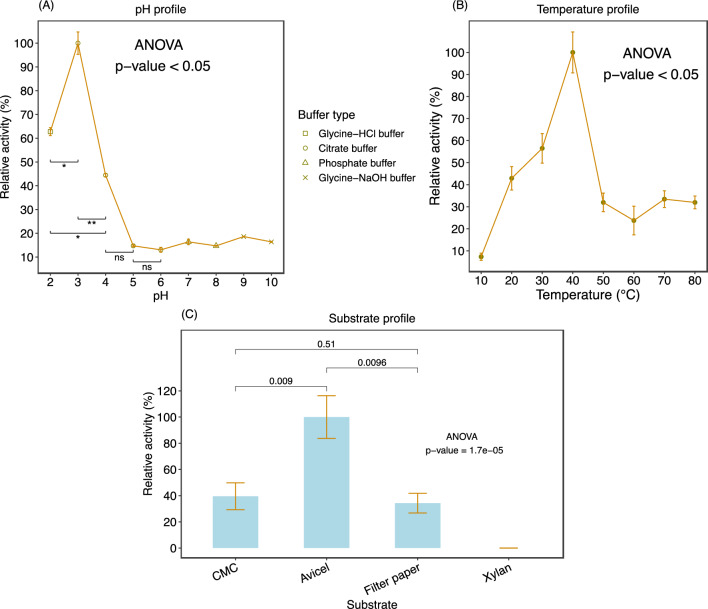
The effect of pH, temperature, and substrate on Celcm05-2 activity. (**A**) Celcm05-2 activity was measured under different pHs ranging from 3 to 10 using different buffer systems. (**B**) The effect of temperature on the CMCase activity of Celcm05-2 was measured in a citrate buffer pH = 3. (**C**) the activity of Celcm05-2 on different polysaccharide substrates. Data are the mean of three independent replications. Percent relative activity was calculated relative to the average activity of the substrate with the highest activity (Avicel). Error bars represent standard deviation. CMC, carboxymethylcellulose.

During the initial screening on a 0.1% CMC agar plate, Celcm05-2 demonstrated efficient degradation of CMC, leading to the formation of a prominent, clear zone (Fig. S1B). To characterize Celcm05-2, the activity of the purified enzyme was assayed under different pH conditions (ranging from pH 2 to pH 10) to determine its optimal pH for CMC degradation. Celcm05-2 exhibited the highest activity at pH 3 and maintained greater than 60% activity at pH 2 and 40% activity at pH 4 (Fig. 4A). The enzyme activity declined significantly (*p*-value < 0.05) at pHs above 4. When assessing the enzyme activity on the CMC substrate under varying temperature conditions (10–80 ℃) while maintaining a constant pH of 3, Celcm05-2 demonstrated its highest activity at 40 ℃ and retained more than 50% of its activity at 30 ℃ (Fig. 4B). Notably, preincubation for 30 h at temperatures of 30 ℃ and 40 ℃ showed that the enzyme could retain more than 50% of its activity. However, a 30-h incubation at 50 ℃ resulted in complete enzyme inactivation (Fig. S2A).

The Celcm05-2 activity was evaluated in the presence of different substrates, including CMC, filter paper, Avicel, and xylan, to determine the substrate specificity of Celcm05-2. The Celcm05-2 displayed a remarkably high level of activity (3.6 U/mL) when Avicel was used as the substrate (Fig. 4C). This suggests that the enzyme has a unique ability to degrade microcrystalline cellulose. However, approximately a twofold reduction in activity was seen when CMC (~ 1.5 U/mL) was used as the substrate. Also, there was a greater than twofold reduction when filter paper (~ 1.3 U/mL) was used as the substrate compared to Avicel.

The kinetic parameters of Celcm05-2 were determined using 1–10 mg/mL CMC as the substrate. K_m_ and V_max_ were estimated to be 0.75 mg/mL and 3.8 µmol min^−1^ mg^−1^, respectively. Notably, Celcm05-2 exhibited a low K_m_ value, indicating a high affinity for its substrate, enabling substantial activity even at low substrate concentrations.

### Effect of metal ions, inhibitors, and organic solvents on Celcm05-2 activity

To investigate the effect of specific metal ions on the CMCase activity of Celcm05-2, two different concentrations (5 and 10 mM) of Ca^2+^, K^+^, Mg^2+^, Mn^2+^, and Ni^2+^ ions were included in the reaction mixture. Ni^2+^, Mn^2+^, and K^+^ ions stimulated Celcm05-2 activity by greater than 50% (Table 1). The highest activity enhancements were observed with 10 mM Ni^2+^ (twofold) and 10 mM K^+^ (1.9-fold). Although 5 mM Ca^2+^ ion improved enzyme activity by 34%, however, the difference was not statistically significant (*p*-value > 0.05). Both Ni^2+^ and K^+^ had a dose-dependent increase in enzyme activity.

**Table 1:** Effect of metal ions and inhibitors on Celcm05-2 activity at different concentrations.

Ions or inhibitors	Concentration	Mean activity ± sd	Relative activity (%)	Comparison	*p*-value
Metal ions					
NiCl_2_	5 mM	1.83 ± 0.1	167.89	vs. control vs. NiCl_2_ 10 mM	*** *
NiCl_2_	10 mM	2.35 ± 0.24	215.9	vs. control	***
CaCl_2_	5 mM	1.47 ± 0.07	134.56	vs. control vs. CaCl_2_ 10 mM	ns ns
CaCl_2_	10 mM	1.16 ± 0.14	106.12	vs. control	ns
MgCl_2_	5 mM	0.82 ± 0.24	75.54	vs. control vs. MgCl_2_ 10 mM	ns ns
MgCl_2_	10 mM	1.11 ± 0.10	101.83	vs. control	ns
MnCl_2_	5 mM	1.87 ± 0.29	171.25	vs. control vs. MnCl_2_ 10 mM	** ns
MnCl_2_	10 mM	1.77 ± 0.04	162.08	vs. control	***
KCl	5 mM	1.67 ± 0.14	152.91	vs. control vs. KCl 10 mM	* ns
KCl	10 mM	2.09 ± 0.21	191.44	vs. control	***
Inhibitors					
EDTA	5 mM	1.15 ± 0.31	78.82	vs. control vs. EDTA 10 mM	ns ***
EDTA	10 mM	0.49 ± 0.03	33.49	vs. control	***
DTT	5 mM	2 ± 0.18	136.67	vs. control vs. DTT 10 mM	** ns
DTT	10 mM	1.85 ± 0.04	126.2	vs. control	ns
PMSF	5 mM	1.62 ± 0.08	110.93	vs. control vs. PMSF 10 mM	ns ns
PMSF	10 mM	1.69 ± 0.03	115.49	vs. control	ns
SDS	0.1%	1.62 ± 0.08	110.93	vs. control vs. SDS 0.3%	ns ns
SDS	0.3%	1.69 ± 0.10	115.49	vs. control	ns
Triton X-100	10%	1.84 ± 0.07	125.97	vs. control vs. Triton X-100 20%	ns *
Triton X-100	20%	2.27 ± 0.24	154.9	vs. control	***

The activity of Celcm05-2 was assessed in the presence of various metal ions and inhibitors at concentrations of 5 mM and 10 mM. Statistically significant differences were determined through One-way analysis of variance (ANOVA) followed by the Tukey post hoc test. Significance levels are denoted as * for *p*-value < 0.05, ** for *p*-value < 0.01, *** for *p*-value < 0.001, and ns for not significant.

The effect of inhibitors including chelating agent EDTA on Celcm05-2 activity was measured at 5 and 10 mM concentrations. The CMCase activity was significantly affected (declined by ~ 70%) by 10 mM EDTA but not the 5 mM concentration (Table 1). The enzyme activity remained unaffected by 5 and 10 mM PMSF and 0.1 and 0.3% SDS. The addition of a 5 mM concentration of the reducing agent DTT stimulated enzyme activity by 36% (*p*-value < 0.01). Furthermore, a 20% concentration (v/v) of the surfactant agent Triton X-100 significantly enhanced enzyme activity by ~ 50% (*p*-value < 0.001).

The effect of a 30% concentration of certain organic solvents, including chloroform, ethanol, methanol, isopropanol, and glycerol, on the CMCase activity of Celcm05-2 was examined. Isopropanol, ethanol, and methanol were found to inhibit the CMCase activity of Celcm05-2 by approximately 10%, 20%, and 30%, respectively (Fig. S2B). Conversely, glycerol and chloroform exhibited an enhancement effect on the CMCase activity of Celcm05-2.

### Biodegradation of waste paper pulp by Celcm05-2

**Figure 5:**
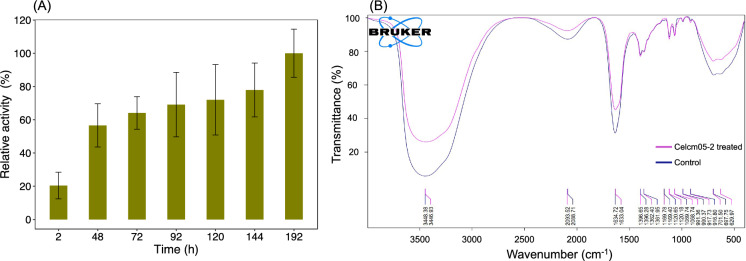
Effect of recombinant Celcm05-2 cellulase enzyme on paper pulp residues. (**A**) Relative activity of Celcm05-2 on paper pulp substrate at 2, 48, 72, 92, 120, 144, and 192 h incubation. Data are means of three independent replications. Error bars represent standard deviation. The activity was normalized to the time point with the highest recorded activity. (**B**) FTIR analysis of paper pulp with (Celcm05-2 treated) and without (control) enzyme treatment. The FTIR spectrum was obtained from paper pulps treated for 192 h.

The waste carton pulps were incubated in the presence and absence of Celcm05-2 for 192 h. The amount of released reducing sugar steadily increased with the incubation time, peaking at 192 h, confirming the hydrolytic activity of Celcm05-2 on paper pulp (Fig. 5A).

The Celcm05-2-treated and control pulp samples underwent FTIR analysis over the 400 to 4000 cm^−1^ wavelength range to study changes in functional groups in paper pulps following enzyme treatment. Both treated and untreated pulp samples exhibited similar absorption patterns, with significant differences observed in band intensities at certain wavenumbers. The most substantial variations in transmittance between the enzyme-treated and control samples were noted at wavenumbers 3446–3448 cm^−1^, 2088–2093 cm^-1^, 1633–1634 cm^-1^, and 629–701 cm^-1^ (Fig. 5B). The band at 3446–3448 cm^-1^ corresponds to the O–H stretching vibrations in the hydroxy groups present in cellulose, hemicellulose, and other polysaccharides within paper pulps^43,44^. A significant increase in transmittance in the Celcm05-2-treated pulp sample in this wavenumber region suggested strong hydrolytic activity of the enzyme, consistent with the DNS-based measurement of the released reducing glucose equivalents (Fig. 5A). The peak in the region 1633–1634 cm^-1^ can be attributed to the stretching vibration of water molecules absorbed in the cellulose^43,45^. The peak at wavenumber 1618 cm^-1^ may also indicate the C = O stretching in aromatic skeletal vibration, characteristic of hemicellulose and lignin typically found in paper pulps^46^. The specific peak at around 697–701 cm^-1^ is attributed to the out-of-plane bending vibration of the C-H bond in the cellulose structure. A significant increase in percent transmittance in enzyme-treated pulp indicates efficient cellulose degradation by Celcm05-2. The band at 629 cm^-1^ in the FTIR spectrum is commonly associated with the bending vibration of Si–O-Si (siloxane)^44^. Siloxane bonds are characteristic of amorphous silica, which can be present in waste carton pulps.

**Figure 6:**
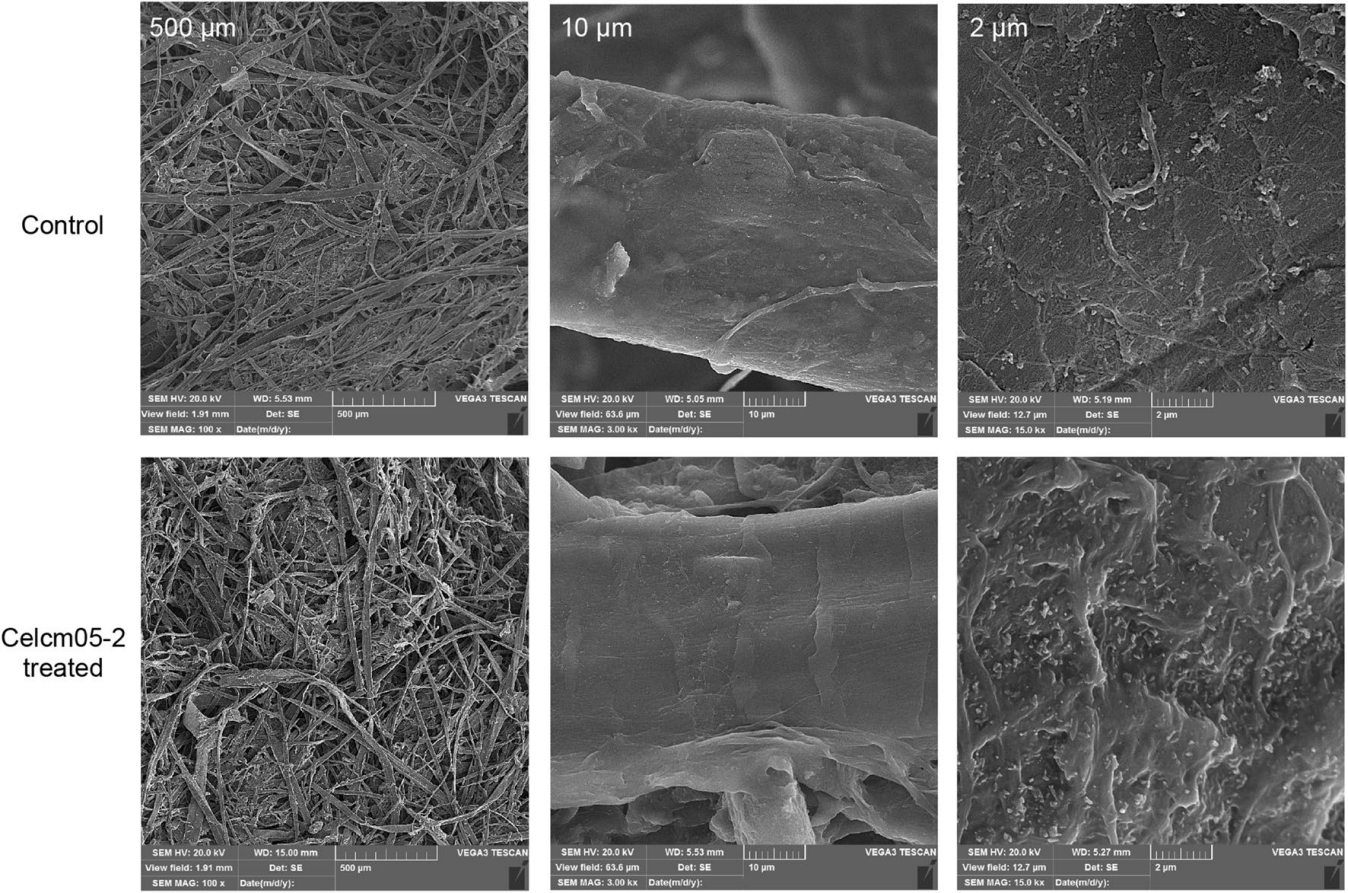
Changes in the morphology of paper pulp residues after treatment with the Celcm05-2. Scanning electron microscopy images show paper pulps treated with Celcm05-2 and a parallel control without enzyme treatment for 192 h. The magnification levels are 500 µm, 10 µm, and 2 µm.

To further visualize morphological changes in the substrate structure upon Celcm05-2 treatment, we analyzed scanning electron microscopy (SEM) images of waste paper pulps with and without enzyme treatment. The images of Celcm05-2 treated pulps revealed clear evidence of enzymatic hydrolysis, indicated by the development of pores, peeling, and loosening of surface structures (Fig. 6). Moreover, evidence of surface polishing was observed at higher magnifications, as the treated material exhibited smoother surfaces with fewer protuberances and flaking. Bio-polishing is a characteristic feature of cellulase enzymes employed in the textile industry.

## Discussion

Metagenome sequencing of microbiota colonizing the GIT of ruminant animals has provided insights into their potential for polysaccharide degradation, opening avenues for enzyme bioprospecting. The rumen microbiota, in particular, synthesizes a wide array of carbohydrate-degrading enzymes that aid in the digestion and utilization of plant cell wall polysaccharides^2,8,13,14^. Recently, efforts have been undertaken to enzymatically characterize specific glycoside hydrolyze enzymes discovered through metagenome sequencing of rumen microbiota, aiming to identify novel enzymes with improved or enhanced catalytic properties^5,9,11^. In this study, we investigated a cellulase enzyme from the metagenome of camel rumen using a combination of in-silico and wet lab experimental assays. The candidate cellulase, named Celcm05-2, was chosen from a list of genes predicted to contain a GH domain from prominent cellulase families deposited in the CAZy database. Phylogenetic analysis identified Celcm05-2 as belonging to a bacterium from the genus *Butyrivibrio*, whose members are renowned for their broad polysaccharide-degrading capabilities^47^.

Structural analysis revealed that Celcm05-2 shares a typical folding pattern found among other GH5 family enzymes, characterized by a distinctive barrel-shaped (β/α)_8_ core structure^42^. Enzymes of this family employ the Koshland two-step mechanism for catalysis, utilizing a pair of glutamic acid residues as the proton donor and the nucleophile^42^. Multiple sequence alignment strongly suggests that these critical residues in Celcm05-2 are Glu236 and Glu368, positioned prominently at the front of the barrel fold. The enzyme’s substrate binding cleft is formed by conserved residues, including Arg145, Asn235, Glu236, His318, Tyr320, and Glu368, which are localized in surface-exposed loops connecting β-sheets and/or α-helices. The His318 residue, positioned between the two catalytic residues (Glu236 and Glu368), facilitates electron transfer between catalytic dyads and thus contributes to the enzyme’s catalytic function^48^. While Celcm05-2 shares significant structural similarities with a GH5 enzyme from *Caldicellulosiruptor* sp. F32 (3D model 4X0V), its substrate profile differs. This implies that structurally non-conserved residues are pivotal in determining substrate specificity.

GH5 enzymes constitute a large family of bi-functional CAZymes that hydrolyze diverse substrates. Characterizing Celcm05-2 for substrate specificity revealed its ability to hydrolyze various typical cellulosic substrates, including CMC, filter paper, and Avicel but not xylan. Notably, the enzyme displayed exceptionally high activity when acting on crystalline cellulose, Avicel, rendering it exceptionally appealing for numerous biotechnological processes requiring the degradation of recalcitrant cellulosic substrates. Unlike most cellulases identified thus far, which prefer soluble cellulose substrates like CMC or amorphous forms of cellulose such as phosphoric acid swollen cellulose (PASC) as substrate^49,50^, Celcm05-2 stands out by its strong performance with crystalline cellulose. This remarkable characteristic is rarely observed in other potential cellulases. Table 2 presents a comprehensive overview of optimal pH, temperature, and specific CMCase and Avicelase activities for cellulases isolated from different microorganisms including bacteria and fungi. Among characterized GH5 endoglucanases, few exhibit appreciable Avicelase activity, with most lacking it entirely or demonstrating activity significantly lower than their CMCase function. A high Avicelase activity of Celcm05-2 can be attributed to its exoglucanase mode of action. Similar results were obtained in other studies, such as with a crude enzyme preparation from *Bacillus sp.* growing on a sugarcane bagasse^51^ or a cellulosome complex from *Bacillus megaterium*^52^.

**Table 2:** Comparing enzymic properties of cellulases characterized from various microorganisms.

Designated name	Source organism	Enzymatic activity	Optimal pH	Optimal temperature (°C)	Avicelase specific activity	CMCase specific activity	Ref
Celcm05-2	*Butyrivibrio* sp.	Cellulase	3	40	3.6 (U/mL)	1.5 (U/mL)	This study
CelA	*Caldicellulosiruptor bescii*	Cellulase	6	75	1 (mU/mg)	9.9 (mU/mg)	^75^
–	*Bacillus*	Cellulase	7.5–8	70	0.83 (U/mL)	0.29 (U/mL)	^51^
ENG1	*Clostridium*	Endoglucanase	7	45	No activity	0.046 (U/mg)	^76^
CelC307	*Cohnella* sp. A01	Endoglucanase	7	40	No activity	13.6 (U/mg)	^77^
–	*Daldinia eschscholzii*	Endoglucanase	6	50	0.45 (U/mg)	6.3 U/mg	^78^
–	*Caldibacillus cellulovorans*	Endoglucanase	6.5–7	80	1.7 (U/mg)	50 (U/mg)	^79^
–	*Botrytis ricini* URM 5627	Endoglucanase	5	50	No activity	0.097 (U/mL)	^80^
–	*Bacillus licheniformis*	Endoglucanase	6	65	No activity	3.1 (U/mg)	^81^
–	*Paenibacillus* sp. strain B39	Cellulase	6.5	60	22.3 (U/mg)	51.94 (U/mg)	^82^
CelE1	Metagenome library	Endoglucanase	7	50	8.8 (U/mg)	13 (U/mg)	^83^
CTendo45	*Chaetomium thermophilum*	Endoglucanase	4	60	–	1.21 (U/mg)	^84^
–	*Bacillus subtilis* strain LFS3	Acidothermophilic cellulase	4	60	No activity	14.42 (U/mg)	^85^
Cel5A	*Basidiomycetes*	Endoglucanase	4.8	50	4.5 (U/mg)	120,000 (U/mg)	^86^
Cel-5A	*Bacillus subtilis* 1AJ3	Endoglucanase	4.5	50	2.11 (U/mg)	19.19 (U/mg)	^59^
DturCelB	*Dictyoglomus turgidum*	Endomannanase/endoglucanase	5.4	70	No activity	0.9 (U/mg)	^87^
CelA	*Bacillus* sp. BP-23	Endoglucanase	4	40	0.007 (U/mg)	2.08 (U/mg)	^88^
TlCel6A	*Talaromyces leycettanus* JCM12802	Thermophilic cellulase	4.5	75	0.12 (U/mg)	0.09 (U/mg)	^58^
cel-1	*Prevotella* sp.	Cellulase	4.5	45	–	6.6 (U/mg)	^6^
celA	*Neocallimastix patriciarum* J11	Cellobiohydrolase	6	50	1.1 (U/mg)	108 (U/mg)	^89^
cel28A	Uncultured rumen bacteria	Endoglucanase	5	50	0.1 (U/mg)	20.56 (U/mg)	^62^
cmgl504	*Cellvibrio* sp.	Endoglucanase	5.5	50	0.003 (U/mg)	15.4 (U/mg)	^90^
EG A/EG B	*Penicillium occitanis* Mutant Pol 6	Endoglucanase	3.5/3.5	60/50	0.018/0.32 (U/mg)	4.67/3.33 (U/mg)	^91^

The values listed in the table are taken from the original publications. Only those cellulases that were tested for both Avicelase and CMCase activities have been included in this table.

Previous research indicated that non-catalytic carbohydrate-binding modules are often necessary for a cellulase enzyme to efficiently degrade substrates like Avicel or filter paper^50,53^. This effect is likely mediated by facilitating enzyme contact with the substrate or exposing amorphous regions of the crystalline cellulosic substrate to the enzyme^53^. However, Celcm05-2 possesses only a single catalytic GH5_4 domain and lacks additional cellulose-binding modules, suggesting that enhanced crystalline cellulose degradation by Celcm05-2 is facilitated through alternative mechanisms. The enhanced Avicelase activity of Celcm05-2 can be attributed to its distinctive structural features, which facilitate efficient accommodation of cellulose microfibrils into the substrate-binding cleft for subsequent hydrolysis. In a GH5_26 family enzyme, for instance, the interaction between a protruded N-terminal region and the residues in the active site leads to a reduced cellulase activity, acknowledging the hypothesis that structural variations can modulate enzyme activity^54^.

The kinetic analysis of CMC hydrolysis by Celcm05-2 revealed a sigmoidal profile and a substantially low K_m_ value, indicating its high efficiency for substrate binding (positive cooperative binding) and catalytic activity. Despite lacking any carbohydrate-binding module, the high affinity for substrate binding suggests the presence of secondary substrate binding sites. In certain CAZymes, these secondary binding sites can compensate for the absence of auxiliary carbohydrate-binding domains that facilitate substrate attachment of the enzyme^55^. Secondary substrate binding sites become particularly crucial when the enzyme functions on large substrates^48^, such as uniformly polymerized materials like Avicel.

Biochemical characterization demonstrated that Celcm05-2 remains active across a narrow pH spectrum, displaying remarkable activity in acidic conditions (pHs 2 and 3). This distinctive feature makes Celcm05-2 a promising candidate for diverse biotechnological applications, including biofuel, laundry, and textiles industries^23,56^. Enzymes isolated from acidophilic microorganisms are known for their ability to maintain activity under low pH conditions^57–59^. While reports of acid-tolerant cellulases exist, they rarely exhibit optimal pH values below 4, as exemplified in Table 2. This scarcity is especially evident within the mesophilic environment of the rumen. This underscores the unique novelty of Celcm05-2 in maintaining activity under extremely acidic pH conditions. Acid-tolerant cellulases are particularly valuable in processes where acid pretreatment of lignocellulosic material is a critical step, such as in producing biofuel from agricultural wastes^60^.

Concerning reaction temperature, the enzyme showed the highest activity at 40 ℃ and maintained over 50% of its activity at 30 ℃. However, it exhibited limited tolerance to high temperatures and lost more than 50% of its activity after 2 h at 50 ℃. The same temperature profile was documented for GH5 endoglucanases isolated from termite hindgut microbiota^48^. Non-covalent interactions between residues in the active site can contribute to the enzyme’s thermostability. The formation of a salt bridge between a pair of Glu and Arg residues in the active site of a GH5 endoglucanase, closely resembling in structure to Celcm05-2, has been demonstrated to enhance thermostability^42^. However, a multiple sequence alignment unveiled alterations in these two residues, preventing the formation of the salt bridge. As a result, this modification could potentially lead to reduced thermal stability in Celcm05-2.

Metal ions can exert various effects on enzyme activity, and elucidating their precise mechanisms of action can be challenging. Some metal ions enhance enzyme activity, while others may hinder enzyme activity by exerting inhibitory effects. Celcm05-2 displayed an improved CMCase activity in the presence of metal ions, including Ni^2+^, Mn^2+^, and K^+^, while maintaining its activity in the presence of Ca^2+^ and Mg^2+^. Its activity also significantly declined in the presence of the chelating agent EDTA indicating that Celcm05-2 is a metalloenzyme. Similarly, enhanced activity was reported for cellulase from *Photobacterium panuliri* in the presence of Ni^2+^ ^61^ and for endoglucanases from the rumen microbiome with Ca^2+^ and Mn^2+^ ^6,62^. The mechanism by which metal ions stimulate cellulase activity is not well understood. However, they may interact with the enzyme and other molecules, serving as electron donors or acceptors, or modifying the enzyme structure^63^. The CMCase activity of Celcm05-2 was also enhanced in the presence of non-ionic surfactant Triton X-100 while preserving its activity in the presence of 0.1% and 0.3% of ionic surfactant SDS. Studies have shown that the stimulatory effect of Triton X-100 on cellulase enzymes can be substrate-specific^64^. Other non-ionic surfactants including Tween-20 and Tween-80 are also known to improve the enzymatic hydrolysis of pure cellulose and crystalline cellulose^65–67^. When applied to pretreated wheat straws, non-ionic surfactants enhanced cellulose hydrolysis by up to 70%, indicating their potential for reducing enzyme requirements in large-scale bioreactors^68^. Surfactants are thought to enhance enzyme activity by stabilizing enzyme structure, influencing enzyme–substrate interactions, and/or modifying substrate structure^69^. Detergent-compatible enzymes, which can retain their activity in the presence of detergents, can be used in laundry and cleaning products.

Investigating the CMCase activity of Celcm05-2 when exposed to diverse organic solvents revealed its capacity to retain over 60% of its activity in the presence of ethanol, methanol, and isopropanol. Notably, it exhibited enhanced activity in the presence of chloroform and glycerol. A cellulase isolated from *Cohnella sp*. A01 was also reported to exhibit increased activity in the presence of glycerol^70^. Similarly, a cellulase from *Bacillus sonorensis* HSC7 exhibited improved CMCase activity in the presence of methanol and chloroform^71^. There are also examples of cellulase enzymes demonstrating improved activity or enhanced stability in the presence of various organic solvents^72,73^. Enhanced enzyme activity in the presence of organic solvents may result from increased flexibility and improved substrate accessibility, stabilization of enzyme structure, or modification of the hydrophobic environment surrounding the enzyme, thereby influencing its catalytic properties. Cellulase enzymes capable of tolerating organic solvents hold particular value in various industrial applications, particularly in low water reaction conditions. These applications include biofuel production, textile processing, fiber modification in the paper and pulp sector, as well as the biocatalytic production of high-value chemicals. For example, our findings highlight the applicability of Celcm05-2 in the biopolishing process, which aims to enhance the quality of cellulosic fibers by diminishing both the pilling inclination and fuzziness observed in knitted fabrics, consequently imparting a smoother surface texture^74^.

## Conclusion

We have successfully cloned and conducted enzymatic characterization of a novel GH5 acidophilic cellulase sourced from the camel rumen microbiota. This enzyme exhibits distinctive attributes, notably its capacity to retain high activity across the acidic pH ranges. This intriguing trait positions Celcm05-2 as a promising candidate enzyme with many potential biotechnological applications. Investigation into its substrate specificity unveiled a broad proficiency in cellulose degradation, with a pronounced efficacy in targeting the crystalline form of cellulose. This trait makes it highly appealing for diverse industrial applications, particularly biofuel production. Moreover, further analysis involving paper pulp treatment underscored its substantial cellulose-degrading ability, highlighting its viability for incorporation into the biopolishing process in the textile industry.

### Supplementary Information

Supplementary Figures.

## Data Availability

The camel rumen metagenome-assembled sequences and the predicted protein-coding genes have been deposited to the Integrated Microbial Genomes (IMG) database under the IMG dataset ID: 3300003523.
