# Supplementary material for: Enhanced crystalline cellulose degradation by a novel metagenome-derived cellulase enzyme
Source: Sci Rep. 2024 Apr 12;14:8560. doi: 10.1038/s41598-024-59256-4 (PMC11014956; doi:10.1038/s41598-024-59256-4)
Supplement: Supplementary file 1 — Supplementary Figures. [file 41598_2024_59256_MOESM1_ESM.pdf]

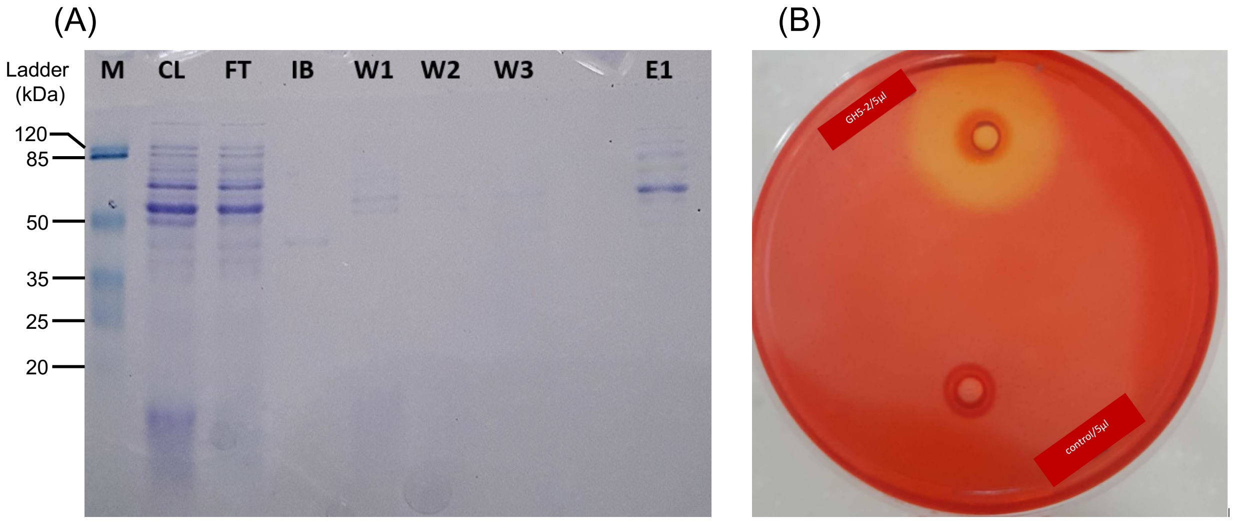

Fig. S1: **Purification and plate assay of the recombinant Celcm05-2 cellulase enzyme.** (A) SDS-PAGE analysis shows the crude cell lysate and the purified enzyme fractions. M, protein size marker (Thermo Scientific, Cat No: 26612); CL: clear lysate; FT: flow-through; IB: inclusion body; W1-3: wash 1-3; E1: protein elute 1. (B) Celcm05-2 activity on an agar plate with 0.1% CMC as substrate. The total protein concentration in the extract was 480 µg/mL.

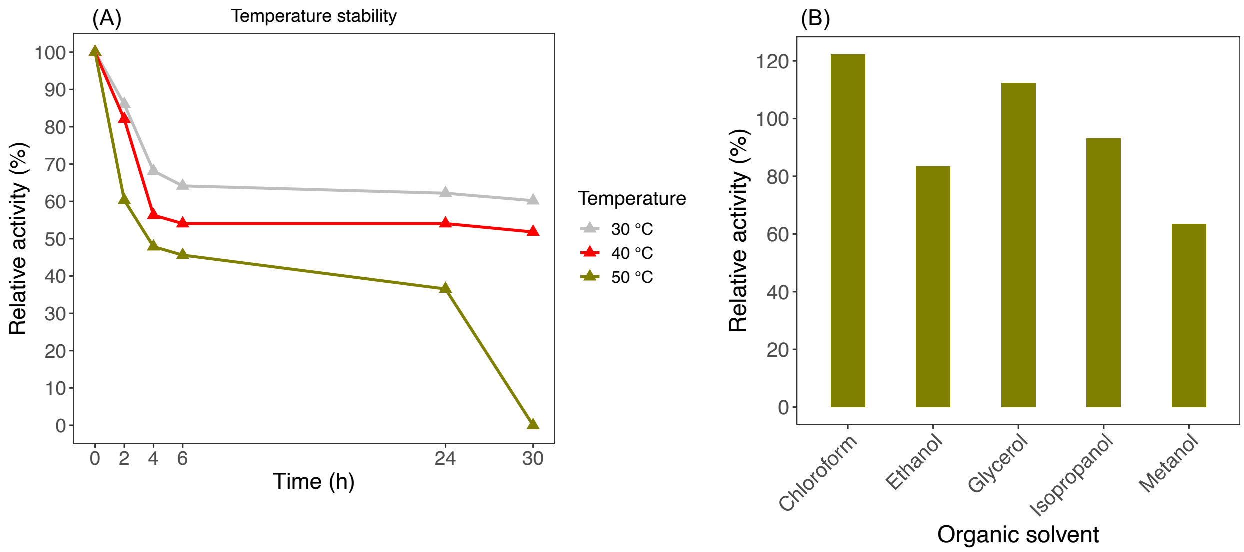

Fig. S2: **The effect of long-term storage and organic solvents on Celcm05-2 activity**. (A) the CMCase activity of the enzyme under optimal temperature (40 °C) and 10 °C below and 10 °C above the optimum temperature. The CMCase activity of Celcm05-2 was measured under standard conditions — citrate buffer pH = 3. (B) The CMCase activity in the

presence of a 30% (v/v) concentration of certain organic solvents was measured under standard conditions.
